# Can Lactoferrin, a Natural Mammalian Milk Protein, Assist in the Battle against COVID-19?

**DOI:** 10.3390/nu14245274

**Published:** 2022-12-10

**Authors:** Alexandra Wilhelmina Carla Einerhand, Carolien Annika van Loo-Bouwman, Gisela Adrienne Weiss, Caiyun Wang, Genna Ba, Qicheng Fan, Baoping He, Gerrit Smit

**Affiliations:** 1 Einerhand Science & Innovation, 1815 JN Alkmaar, The Netherlands; 2 Yili Innovation Center Europe, 6708 WH Wageningen, The Netherlands; 3 Inner Mongolia Dairy Technology Research Institute Co., Ltd., Hohhot 010110, China; 4 Inner Mongolia Yili Industrial Group Co., Ltd., Hohhot 010110, China

**Keywords:** lactoferrin, nutraceutical, food, dairy, COVID-19, coronavirus, SARS-CoV-2, anti-viral, immunomodulation, iron-binding

## Abstract

Notwithstanding mass vaccination against specific SARS-CoV-2 variants, there is still a demand for complementary nutritional intervention strategies to fight COVID-19. The bovine milk protein lactoferrin (LF) has attracted interest of nutraceutical, food and dairy industries for its numerous properties—ranging from anti-viral and anti-microbial to immunological—making it a potential functional ingredient in a wide variety of food applications to maintain health. Importantly, bovine LF was found to exert anti-viral activities against several types of viruses, including certain SARS-CoV-2 variants. LF’s potential effect on COVID-19 patients has seen a rapid increase of in vitro and in vivo studies published, resulting in a model on how LF might play a role during different phases of SARS-CoV-2 infection. Aim of this narrative review is two-fold: (1) to highlight the most relevant findings concerning LF’s anti-viral, anti-microbial, iron-binding, immunomodulatory, microbiota-modulatory and intestinal barrier properties that support health of the two most affected organs in COVID-19 patients (lungs and gut), and (2) to explore the possible underlying mechanisms governing its mode of action. Thanks to its potential effects on health, bovine LF can be considered a good candidate for nutritional interventions counteracting SARS-CoV-2 infection and related COVID-19 pathogenesis.

## 1. Introduction

The human coronavirus SARS-CoV-2, causing coronavirus disease 2019 (COVID-19), has rapidly spread around the globe since its discovery in December 2019. In March 2020, the World Health Organization (WHO) has declared the novel coronavirus outbreak a global pandemic. Up until today, SARS-CoV-2 has killed more than 6.6 million people and infected at least 642 million individuals worldwide while the numbers are still increasing [1]. In most countries, the COVID-19 pandemic has overloaded healthcare services and has led to huge economic losses. Clinical symptoms of COVID-19 vary from asymptomatic infection to severe lung failure requiring hospitalization and mechanical ventilation [2]. SARS-CoV-2 transmission typically occurs through lung droplets and the incubation period is about 6.6 days [3]. The most common symptoms of COVID-19 include dry cough, fever, shortness of breath, muscle pain and fatigue [4,5]. About 30% of patients have long-term symptoms after a SARS-CoV-2 infection persisting longer than a month after COVID-19 [6]. This is called ‘Long COVID’. Around 18% of COVID-19 patients suffer from gut-related complaints like diarrhea, nausea/vomiting, or abdominal pain [7].

Since early 2020, nutrients and bioactive compounds have been studied for potential roles in prevention and/or complementary treatment of disease symptoms [8,9,10] alongside the effect of nutrient deficiencies (e.g., vitamins C, D, E and selenium and zinc) affecting the severity of COVID-19 [11,12]. One of these bioactives has shown consistent results both in vitro and in vivo: the milk-derived bioactive protein lactoferrin (LF) [13].

LF is a naturally occurring, multi-functional glycoprotein with a binding property for iron. It is present in different body fluids including most prominently human and animal milk, but also nasal and bronchial secretions, gastrointestinal fluids, urine mucosal secretions, saliva, tears, and in granules of leukocytes. Since LF was discovered in 1939 [14], it has been connected with the body’s natural defense system. As a known part of the native immune system it performs an essential role in the first line of defense against viral and microbial infections. 

LF is one of the most abundant whey proteins in breast milk. A high concentration of LF is found in human colostrum, the initial milk produced immediately post-partum. Concentrations gradually drop from 8 g/L in colostrum to 3.5–4 g/L in mature breast milk [15,16,17,18,19]. Of the bioactive proteins present in breast milk, LF appears to be a crucial player performing wide-ranging functions protecting the newborn against infection [20]. LF is related to successful development of the infant’s immune system [21], it chelates iron and has direct anti-viral and anti-microbial effects [19]. Its beneficial effects have been particularly well-studied in newborn infants to investigate its role in the prevention of sepsis and necrotizing enterocolitis [22]. This is not only due to the fact that LF is present in breast milk, but also because LF is produced in human native immune and epithelial cells, and is excreted in the blood [21]. The concentration of LF in blood is normally low, but this can increase up to 1000 times at sites of inflammation due to its release from neutrophils [23,24]. In bovine milk, the concentration of LF changes from 2–5 g/L in colostrum to 0.1–0.3 g/L in mature bovine milk [25,26].

LF sources other than human are available, because homology between bovine and human LF is 69% and the proteins have a very similar structure [27]. LF derived from bovine milk has wide applications in tablets, supplements and fortified foods like formulas, dairy products and beverages [28]. LF might be regarded as an ideal nutraceutical because sourcing from bovine milk is relatively cheap, it is well-tolerated after ingestion, plus the protective activities have been well demonstrated. The key protective activities demonstrated by supplemental bovine LF are highlighted in this paper. Both bovine and human LF have received increased attention lately due to their potential role against COVID-19. To date most of the intervention studies are carried out with bovine LF, a food ingredient that is generally recognized as safe to use in (medical) food by many authorities around the world like China, EU and US. Unless otherwise stated LF refers to bovine LF.

The aim of this narrative review is both to summarize the previous (pre)clinical studies on the bioactive characteristics of LF within the context of potential applications against SARS-CoV-2 and to investigate the potential underlying mechanisms governing its course of action. Furthermore, we propose the possibility of supplemental LF as a potential candidate for nutritional interventions against SARS-CoV-2 infection and related COVID-19 pathogenesis.

## 2. COVID-19: A Continuous Burden on Mankind

### 2.1. Pathogenesis

COVID-19 following SARS-CoV-2 infection can be categorized in an early and a late phase. In the early phase mainly virus replication and virus-mediated cell damage play a role. The immune responses come in during the late phase, in which the cells initiate an inflammatory response with the recruitment of T lymphocytes and the secretion of various cytokines. COVID-19 creates a ‘cytokine storm’ in which local and systemic hyper-inflammation play a major role in the disease process. This also leads to hyper-coagulability with vascular complications [29]. SARS-CoV-2 can also affect other organs, like the gastrointestinal tract (in 18% of cases), heart, brain, bone marrow, liver and kidneys [30,31].

SARS-CoV-2 can enter host cells in the early phase of the disease through the receptor binding domain subunit of the spike protein. The virus is transmitted through airborne lung droplets or by direct contact. Entering the human body via the nose, mouth or eyes, the virus spreads to the back of the nose. There it attaches to and enters the epithelial cells via specific cell surface receptors. One of these receptors is the angiotensin-converting enzyme 2 (ACE2) with the host transmembrane protease serine 2 (TMPRSS2) promoting the entry of the virus into the cell [32,33,34]. Meanwhile, the SARS-CoV-2 is also recognized by pattern-recognition receptors on immune cells responsible for initiating the host defense system and production of inflammatory cytokines [35]. Subsequently, the virus spreads from the nose to the pharynx and bronchus, eventually entering the lower parts of the lungs where it infects alveolar epithelial type 2 cells leading to acute respiratory distress syndrome (ARDS). Prominent early characteristics of COVID-19 include severe hypoxia and a marked reduction in B cells, the latter being vital in defense against SARS-CoV-2 [36]. Nearly 20% of all COVID-19 patients progress to the stage involving the lower part of the lungs and develop severe symptoms [37]. When the virus infects and replicates in the alveolar lung cells, it triggers intense inflammation and induces oxidative stress causing damage to the cell walls of the alveoli with leakage of fluid that fills up these alveoli [38]. In this situation the immune system may over-react sending in neutrophils, T-helper- (CD4) and cytotoxic T-cells (CD8) that release pro-inflammatory cytokines, especially IL-1 and IL-6 [37,38]. This hyper-inflammation termed ‘cytokine storm’ unfortunately inflicts more damage on the healthy lung cells than on the targeted virus. In addition, the virus attacks hemoglobin causing iron release into the circulation. Thereby hemoglobin fails to bind with oxygen and hinders its delivery to major organs resulting in rapid multi-organ failure [39]. 

Currently, COVID-19 treatment is mostly supportive. However, early results of several anti-viral agents (e.g., remdesivir) and immunotherapy with steroids (e.g., dexamethasone) and various cytokine inhibitors (e.g., anti-IL1, anti-IL6, anti-IFN) seem promising in showing efficacy directly against SARS-CoV-2 itself [2,35]. Also, effective vaccines have been established, which helps curb the pandemic. To date, almost 12 billion vaccines have been administered globally [1]. However, new variants of SARS-CoV-2 constantly pop up and remain to be a key threat to people across the world. The first vaccines, based on the SARS-CoV-2 variant that emerged December 2019 in Wuhan, China, do not work well in protecting against the current Omicron strains although they do seem to protect against severe disease. In August 2022, the Food and Drug Administration (FDA) in the USA and the European Medicine Agency (EMA) authorized two new vaccines (from Pfizer-BioNTech and Moderna) designed to protect against disease caused by the original SARS-CoV-2 virus strain as well as the Omicron variant. These vaccines are extremely effective against severe disease, hospitalizations, and death from the specific variants, but none of the vaccines is 100% effective. Additionally, vaccinated people that are infected could still spread the virus to other people, although likely they will be infectious for a shorter time.

### 2.2. COVID-19 in the Aging Population

Seniors (65+), nowadays representing 10% of the world population, are the most susceptible to COVID-19. Moreover, underlying age-related health concerns, such as diabetes and hypertension, are risk factors that can lead to additional complications during infection [40]. Aging but also obesity, smoking and malnutrition weaken the immune system and are thus associated with increased risk of infection resulting in severe disease [9]. According to a study in Wuhan, where the outbreak started, the prevalence of malnutrition is elevated in seniors with COVID-19 [11]. 

During aging, the immune system weakens due to processes called immuno-senescence and inflammaging. Immunosenescence is an ongoing accumulation of senescent immune cells slowing down the clearance of pathogens, whereas inflammaging is a low grade inflammation happening during aging causing immune dysfunction [41]. Recent data from COVID-19 studies indicate that immunosenescence and inflammaging are key drivers of the high mortality rates in seniors [42].

**Figure 1:**
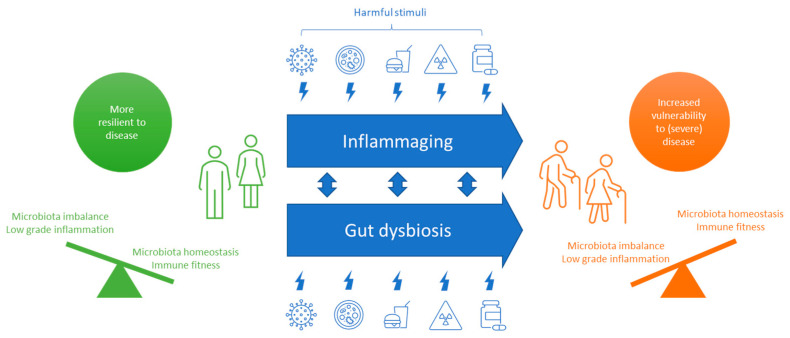
With age, the human body becomes less resilient and more vulnerable to (severe) disease due to inflammaging and gut dysbiosis. In healthy adults the immune system is fit, with the pro- and anti-inflammatory processes in balance, and the beneficial and pathogenic microorganisms in balance. The body is resilient to disease. During aging, the body has to respond to all kinds of harmful stimuli including viruses, pathogenic bacteria, a poor diet, toxins, and drugs, leading to inflammaging and gut dysbiosis; shifting the balance towards higher vulnerability to diseases like COVID-19, heart disease, diabetes, cognitive decline and frailty.

A range of harmful stimuli over a lifetime can lead to inflammaging, including viruses, pathogenic microorganisms, a poor diet, mental stress, toxins, specific drugs or a sedentary lifestyle [43]. People have to constantly adapt to these harmful stimuli and with age people tend to have a more sedentary lifestyle. As a result, the immune balance may shift to a more pro-inflammatory state and age-linked disorders may emerge and an inability to respond effectively to viruses and other pathogens. Adding a SARS-CoV-2 infection to the inflammaging process is like pouring fuel on a burning fire, resulting in hyper-inflammation, a cytokine storm, and severe disease (Figure 1).

Performance of the immune system is strongly linked to performance of the gut microbiome and the other way around [44], because the gut houses over 70% of the body’s immune cells. With age, the number of beneficial microbes in the gut declines, the microbiome is less diverse and disbalanced due to external triggers like viruses, a poor diet, pathogenic microorganisms and drugs (e.g., antibiotics) [45,46,47]. This dysbiosis influences the native immune response and may lead to inflammaging, an inability to protect against pathogens and digest food properly, and to unhealthy aging [45]. Therefore, one can imagine that a gut dysbiosis might increase the possibility of a SARS-CoV-2 infection. In COVID-19 patients, the composition of the gut microbiome was shown to be different [48,49]. The number of beneficial bacteria like *Faecalibacterium prausnitzii* was correlating negatively with COVID-19. Furthermore, gut dysbiosis alongside gut inflammation raises levels of ACE2, a cell surface receptor targeted by SARS-CoV-2 increasing the risk of infection [35,36]. Figure 1 graphically summarizes the proposed aging processes that lead to vulnerability to (severe) diseases like COVID-19.

In the battle against COVID-19, lifestyle changes can counteract inflammaging and reduce both chronic inflammation and elevated cytokine levels. Physical activity especially in combination with dietary modifications reduces inflammatory markers rapidly [50]. In this context, specific nutrients have gained plenty of attention during this pandemic as well-tolerated and cheap alternatives to drugs in order to prevent or fight disease without inducing any adverse effects. Especially, LF, a mammalian milk protein, could be a powerful tool to support health by counteracting infection, balancing the immune system and stimulating a healthy microbiota and epithelial barrier function.

## 3. Lactoferrin’s Protective Effects against SARS-CoV-2 Infection

**Figure 2:**
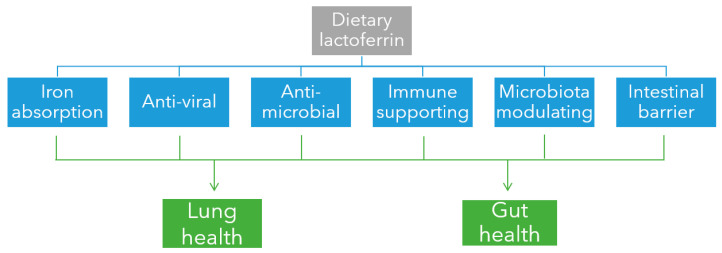
Physiological effects of LF (Blue) that support health of the 2 most affected organs in COVID-19 patients (Green).

LF has been intensely studied over the last decades and is viewed as a key protein to fight infection, aid the immune system, and circumvent iron deficiencies [20,21,28,51,52,53,54,55,56,57,58,59,60,61]. A PubMed search (accessed 1 August 2022) revealed over 120 published clinical trial papers of which the majority (>105 trials) focused on anti-viral, immune or iron-related physiological effects of LF. However, LF also has been shown to fulfil anti-microbiological, microbiota- and intestinal barrier-related functions (Figure 2). In this narrative review the focus is on these six physiological effects of LF that especially support health of the 2 most affected organs in COVID-19 patients, the respiratory and the gastrointestinal tract. Since 2020, LF has been extensively studied in relation to SARS-CoV-2 infections leading to 43 original, mostly preclinical and some clinical study publications.

The physiological effects of LF intimately depend on the tertiary structure of LF. LF is mainly extracted from bovine milk and used in numerous commercial products such as infant formula, nutritional supplements, and functional foods. LF is sensitive to denaturation induced by temperature and other physicochemical stresses like high pressure and drying. Recently several reviews have appeared highlighting that extraction and powder formation processes of LF-containing products have to be optimized, like avoiding/minimizing heat treatment and drying, to minimize its undesired denaturation [62,63].

### 3.1. Iron-Binding and Absorption

Iron deficiency anemia affects nearly 20% of the world population [64]. It may also play a major role in multiple organ dysfunction syndrome in COVID-19 as the virus damages hemoglobin thereby releasing iron into the bloodstream causing oxidative stress and cell damage [39,65]. A meta-analysis of 189 studies involving more than 57,000 COVID-19 patients across all ages, showed an increased amount of ferritin, an iron-binding protein, among COVID-19 patients with severe symptoms compared to moderate cases, and in non-survivors versus survivors [66]. Ferritin is known to play a critical role in inflammation by contributing to the development of a cytokine storm [66]. Iron deficiency, and elevations in serum ferritin can persist for around 2 months after the onset of COVID-19 in some patients [67]. Viral replication depends on host cell iron enzymes, some of which are involved in transcription, viral mRNA translation, and viral assembly [68]. SARS-CoV-2 infection induces a pro-inflammatory cytokine storm, including IL-6 [69], which in turn dysregulates iron homeostasis leading to an intra-cellular iron overload [70]. Such an iron overload increases viral replication, thereby enhancing the seriousness of the infection [71]. Collectively, these data highlight the potential involvement of iron and related proteins in COVID-19 pathology.

LF is an iron-binding protein belonging to the transferrin family of proteins. One of its main physiological effects is related to iron absorption [72]. Usually, LF is only partially saturated and has an iron saturation of about 15–20%. Apo-LF is iron-depleted LF (<5% iron saturation) whereas saturated LF is known as holo-LF [39]. Thanks to its ability to bind iron, it plays an antioxidant role within the body. In cases of infection and excessive iron in the body, there may be overproduction of reactive oxygen species (ROS), leading to oxidative stress and causing significant cell damage. LF disrupts the production and elimination of these ROS by preventing oxygen and iron from binding [73,74,75]. 

LF-bound iron present in milk and other dairy products becomes available to the human body through the intestinal uptake of LF via the LF receptor, making LF a nutritional iron source with similar or even better efficiency as inorganic iron salts. Via this route, LF can confer protection against anemia, especially in populations at risk of iron deficiency like COVID-19 patients [76,77,78,79]. In addition to the well-characterized iron-binding activities, LF has been shown to modulate expression of major iron proteins, such as ferritin and ferroportin, in preclinical studies as well as in human intervention trials [70,80]. In COVID-19 patients, early oral administration of LF decreases serum ferritin levels [69]. As iron and iron-related proteins play a role in COVID-19 pathogenesis, LF might be of interest as a nutraceutical agent. Furthermore, apo-LF avidly can bind iron, making it unavailable to SARS-CoV-2 that requires iron for viral replication and for its functions [81]. Therefore, iron chelation therapy using LF in COVID-19 has been suggested to be an additional approach to arrest viral replication with the prerequisite of adequate understandings on the patient’s iron status, including iron, ferritin, and hemoglobin levels (Table 1) [81,82].

### 3.2. Anti-Viral Activity

Although LF has several biological benefits, the host-protective effects against pathogens including viruses, bacteria, and fungi are regarded as one of its most beneficial [83]. Several reviews have highlighted the in vitro anti-viral effects of LF against pathogens that cause common infections such as influenza, the common cold, summer cold, gastroenteritis, polio, and herpes. In these cases, LF inhibits mainly viral attachment or entry into the target cells [29,53,83,84]. Lately also the number of in vivo studies indicating the protective effects of LF against common viral infections including SARS-CoV-2 have increased [29,85]. A recent meta-analysis of 6 LF intervention studies in infants (4 studies) and adults (2 studies) reported a significant risk reduction of developing respiratory tract infections when using LF (dosage range in adults from 200–2000 mg/d) [86]. Four independent randomized trials in infants from China, Japan and the US demonstrated that LF in infant formula is a promising intervention to prevent acute respiratory tract illness or infection [87,88,89,90]. In adults, LF was studied in relation to direct measures on viral infectious diseases as a common cold and a summer cold. In the study of Vitetta et al., a daily combination of 400 mg LF and 200 mg Ig-enriched whey protein was given to individuals that frequently suffer from cold episodes. This 90-day intervention significantly reduced the number of cold episodes [91]. Although results are promising, it is not yet clear if LF alone would have similar effects on common cold. However, another study investigated the effect of LF alone on infectious disease in the summer season in Japan. To investigate this, doses of 200 mg and 600 mg of LF and a placebo were administered to healthy Japanese adults for 12 weeks in a double-blind study [92]. Although the prevalence of infectious diseases, including summer colds, were not significantly different, the duration of total infectious diseases and in particular that of the summer cold were significantly shorter than in the placebo group. In summary, in adults LF in combination with Ig-enriched whey protein may affect the number of common colds, whereas LF alone (200 mg) may affect the duration of infectious diseases (especially summer colds) in a dose-dependent manner.

In addition to viruses causing respiratory tract infection, LF targets other viruses causing gastroenteritis like norovirus, rotavirus and enterovirus [83,85]. Norovirus is an important pathogen that causes a majority of gastroenteritis outbreaks worldwide across all ages. So far, three independent studies were conducted to analyze the effects of LF against norovirus. Surprisingly, these studies were all done in children. The oral administration of LF at a dosage of 0.5 g/day for 6 months in norovirus-infected young children (12–18 months) has led to a decrease in duration and severity of gastroenteritis-related symptoms compared to placebo, but no reduction in diarrhea incidence is reported [93]. In another study, the daily oral administration of LF to children reduced the incidence of noroviral gastroenteritis [94]. Lastly, a survey in nursery school children consuming 100 mg LF-containing products including yogurt and dairy drinks indicated a lower incidence of norovirus-like gastroenteritis in children who regularly consumed LF products compared to the control group [95]. Because to date there is no adequate treatment for noroviral gastroenteritis, LF seems a promising candidate to help prevent infection, and further studies, especially also in adults, are warranted to establish more reliable evidence.

Rotavirus and enterovirus infections have been analyzed in three studies comparing the effects of LF administration at a daily dosage between 70 and 100 mg versus placebo [83,85]. In a twelve-week study, 100 mg/day LF reduced the severity of rotaviral gastroenteritis although there was no significant benefit in reducing infection incidence. The addition of recombinant human LF and lysozyme (both derived from a recombinant rice [96]) to a rice-based oral rehydration solution reduced the duration of acute diarrhea in children whose rotavirus was identified as a pathogen [97]. In young children receiving 70 mg/day LF over 1 year in a day care setting, no differences in the prevention of enterovirus or rotavirus infection or serum IFN-γ and IL-10 were observed [52]. In children receiving 100 mg/day in a day care setting, absences due to vomiting were reduced [52]. In summary, LF doses of minimally 100 mg per day seem effective in reducing gastroenteritis-related symptoms as a result of viral infections.

Based on the abovementioned extensive antivirus properties of LF against a wide range of common viruses, it can be hypothesized that LF may be used as a potential nutraceutical/food ingredient for the prevention and/or adjunct treatment of COVID-19. Recent evidence indeed suggests that LF may have such potential by inhibiting virus attachment, internalization and replication (Table 2) [29,53]. 

In addition to ACE2, heparan sulfate proteoglycans (HSPGs) can be recognized by SARS-CoV-2 as a receptor for cell attachment [98]. Binding of the viral spike proteins to HSPGs leads to virus enrichment on the cell surface facilitating the subsequent binding with ACE2. In vitro, LF can prevent SARS-CoV-2 infections by blocking the interaction between the virus and HSPG receptors on the cell surface in an ACE2-independent fashion [98,99]. Via this mechanism LF significantly interferes with viral anchoring, preventing high viral concentration on the cell surface, as well as the contact with the specific entry receptor, namely ACE2, which would result in full infection. 

After the initial contact with the host cells via HSPG, SARS-CoV-2 then rolls onto the cell membrane and scans for ACE2, its specific receptor, to bind and lead subsequent cell entry. ACE2 is well expressed in the epithelial cells of the nose, providing an important point of entry for SARS-CoV-2, whereas ACE2 expression in the lower lung is restricted to alveolar epithelial type II cells. This difference in ACE2 expression level in the respiratory tract is mirrored by the SARS-CoV-2 infection gradient, with nasal epithelial cells being primary targets for SARS-CoV-2 replication in the early stage of infection [100]. In addition to the nasal cavity, also the mouth may be an important initial entry point as the spike protein of SARS-CoV-2 binds to ACE2 in salivary glands and SARS-CoV-2 has been consistently detected in the infected patient’s saliva [101,102]. ACE2 is also expressed on other cell types, such as in the esophagus, ileum, myocardium, kidney and urothelial cells at least in part explaining why the infection often is not limited to the lungs [30]. Tissue tropism is dependent on the SARS-CoV-2 variant as the SARS-CoV-2 Omicron variant has a tissue tropism towards the upper respiratory tract [103]. 

LF has been shown to inhibit virus infection of a range of different SARS-CoV-2 variants including the Delta variant, one of the most virulent and potentially more deadly virus strains [104,105]. LF seems more potent than human LF [104,105]. The effects seem to be correlating to LF itself or its proteolytic product lactoferricin B (residues 17–41) but not to other dairy proteins in whey because Wotring et al. did not observe in vitro efficacy for the latter samples against SARS-CoV-2 [104]. When there was efficacy for a sample, it was correlated with the fraction of LF, suggesting that the anti-viral activity was from LF alone or its proteolytic product lactoferricin B, which is the *N*-terminal positively charged region in LF [104,105]. It is of note that the iron-binding property of LF had no effect on the early stage of virus binding to the host cells in vitro [104], indicating that the iron-chelating property of LF affects mainly virus replication and inflammatory steps thereafter.

LF can affect the replication of the virus, because the anti-viral activity of LF is synergistic with the anti-viral drug remdesivir in cell culture [105]. Furthermore, LF combined with diphenhydramine, an antihistamine used for allergy symptoms, can reduce the replication of the SARS-CoV-2 by 99% in vitro suggesting that LF in vivo may shorten the recovery time [106,107]. Individually, LF and diphenhydramine each inhibited SARS-CoV-2 virus replication by about 30% in vitro [106]. This may be due to the fact that in COVID-19 patients, the virus “hijacks” stress-response machinery, including sigma receptors, in order to replicate in the body. Interfering with that signaling appears to be the key to inhibiting the virus’s potency. Data from the experiments show that combinations of highly specific sigma receptor binding products, such as diphenhydramine and LF have the potential to prevent virus infection and decrease recovery time from COVID-19 [106]. Thus, anti-viral effect of LF against SARS-CoV-2 variants is at least in part mediated through preventing the virus from binding to the target cell surface and inhibiting virus replication, which would be predominantly effective during the early phase of the virus infection in the salivary glands and throat when given orally, and in the nose and upper respiratory tract when given as a nasal spray [83]. Orally supplemented LF might also have potential in the more distal parts of the gastro-intestinal tract, as SARS-CoV-2 is known to also cause gastro-intestinal complaints in 18% of COVID-19 cases [7].

### 3.3. Anti-Microbial Activity

Xu et al. [108] recently mapped the research hot spots and development trends regarding the antibacterial effect of LF. Based on this analysis, it is clear that the development of LF as a natural antibacterial is a rapidly evolving research area [108]. The COVID-19 outbreak indirectly led to an annual increase in the number of publications. 

LF has a strong affinity to iron that is essential for cell growth and proliferation. Thanks to its capacity to sequester iron, it deprives microbes of this essential element for their growth and development [23]. It therefore has recognized anti-microbial properties (Table 3). It is actually the first discovered and one of its most well-known characteristics of LF [109]. LF reduces the growth of a wide variety of microorganisms, including gram-negative and gram-positive bacteria, fungi, and protozoa [57,110,111,112]. It effectively inhibits the growth of *Candida tropicalis*, *Escherichia coli*, *Helicobacter pylori*, *Legionella pneumophila, Staphylococcus aureus*, *Salmonella typhi*, *Streptococcus* and *Trichomonas vaginalis*. 

In addition to the bioactivity of intact LF, studies have shown that some peptides formed from LF also have potent activity against specific pathogenic bacteria whereas they do not inhibit specific beneficial bacteria like bifidobacteria and lactobacilli [113]. The latest research showed LF’s anti-fungal effect against pathogenic *Streptomyces scabiei* due to its short bioactive peptides, and LF might be more effective than human LF [114].

LF and LF-derived peptides probably kill bacteria in 4 different ways: 1. The high iron affinity limits iron availability to microorganisms [115]. 2. It also specifically interacts with lipopolysaccharides in the cell membranes of specific microbes, thereby causing fatal damage [116]. 3. The interaction of LF with the bacterial membrane also induces the activities of other antibacterial factors, including lysozyme [84]. 4. Finally, LF can exert antibacterial activity by inhibiting enzyme function [117]. 

Many in vivo preclinical studies showing anti-microbial effects have been reviewed by Teraguchi et al. [118]. The promising results obtained in these preclinical models led to clinical trials. Especially the role of LF in human milk and infant formulas has much clinical evidence [119]. Currently, LF is used as an additive ingredient in various foods, such as infant formula, but also yogurt, skimmed milk, and beverages. In vivo, LF added to formula milk or directly supplemented can prevent sepsis in high-risk preterm infants [22]. Preterm infants are at risk for sepsis causing high mortality and morbidity despite treatment with antibiotics. A recent meta-analysis of 12 controlled trials showed that LF supplementation decreased late-onset sepsis and shortened the hospital stay, indicating that LF may act against certain pathogens in these preterms [22].

In adults, Okuda et al., confirmed the antibacterial activity of LF in inhibiting colonization by *Helicobacter pylori* in humans [112]. This is one example of the applications of LF as an anti-microbial agent in humans. Various other studies have demonstrated that oral administration of LF can reduce bacterial and fungal infections in the gut [120,121].

According to a systematic review, the majority of patients with COVID-19 received antibiotics (71.9%) to prevent bacterial co-infections [122]. Bacterial co-pathogens are usually commonly identified in viral respiratory infections and are important causes of morbidity and mortality. However, in a meta-analysis of 24 studies the overall prevalence of bacterial infection in patients infected with SARS-CoV-2 was 6.9%, whereas in critically ill patients it was more common (8.1%) [122]. Secondary bacterial infection happened in 14.3% of patients. The authors of the meta-analysis concluded that the majority of these patients may not require empirical antibacterial treatment [122]. To date it is unclear how many of these patients consumed LF-containing foods or supplements. LF’s antibacterial effect may still be relevant for these patients to prevent these co-infections and/or secondary infections or support treatment. However, this needs further clinical substantiation. 

It is of note that LF, as a naturally occurring protein in saliva, is supposed to provide microbial homeostasis in the oral cavity [123]. Aging is known to affect the composition of the oral microbiome causing dysbiosis, increased infections, and persistent low-grade inflammation, which may ultimately compromise overall health [124]. In saliva, LF expression is affected by an inflamed oral mucosa that can contribute to occurrence of an oral dysbiosis [125]. Furthermore, LF is expressed at a lower level in saliva as we age [126], making elderly more vulnerable to (infectious) diseases like COVID-19. The use of food products with LF like dairy products increases the level of LF in the oral or nasal cavity, thus strengthening the first line of defense against pathogenic bacteria and viruses [123].

### 3.4. Immune Modulation

Known as a natural antibiotic and anti-viral agent, LF is also an important component that bridges the native and adaptive immune systems of mammals and plays roles in protecting human cells at all stages of life [21,123,127,128,129]. With its positively charged *N*-terminus, LF can bind to negatively charged cell surfaces (e.g., proteoglycans) thereby regulating the native and adaptive immune responses and influencing the expression of pro- and anti-inflammatory cytokines [21]. Preclinical studies demonstrated a role of LF in affecting the expression of several chemokines (e.g., IL-8), anti-inflammatory (e.g., IL-10) and pro-inflammatory cytokines (e.g., TNF-α, IL-12) [21]. For instance, in colostrum-deprived neonatal piglets receiving a formula enriched with LF, the in vivo IL-10 production was increased in spleen [130]. After LF treatment in an experimental colitis model, pro-inflammatory factors (TNF-α, IL-1β, and IL-6) significantly decreased, while anti-inflammatory factors (IL-10 and TGF-β) were maintained or even enhanced [131].

The effects of administration of LF on the cytokine response in humans was reported in several studies. In a non-blinded study, Ishikado et al. reported an increase of the anti-viral IFNα in healthy volunteers with 319 mg/d liposomal LF [132]. In post-menopausal women, given 250 mg/d LF, the expression of pro-inflammatory markers (IL-1β, TNF-α, IL-6, IL-12, and C-reactive protein) decreased while the anti-inflammatory IL-10 increased [133] (Table 4). In pregnant women, affected by anemia of inflammation, 200 mg/d LF administration decreased IL-6 and increased hematological parameters [70,80]. 

LF also affects the adaptive immune system by promoting: (1) the maturation of T-cell precursors into competent helper cells, and (2) the differentiation of immature B-cells into antigen presenting cells [21,134] (Table 4). Clinically, Mulder et al. reported effects on cells from the adaptive immune system [73]. With a dosage of 200 mg/d LF a significant increase in total T-cell activation, T-helper cell activation and cytotoxic T-cell activation was reported. Furthermore, LF knock-out mice have a deficient B-cell and intestinal development, and are more susceptible to periodontitis and experimental colitis [135,136].

Many genes involved in the innate immune response, including endogenous LF, may participate in SARS-CoV-2 clearance. LF is highly elevated (up to 150 folds) in SARS-CoV-1 patients in comparison with healthy volunteers and influenza virus infected patients [137,138]. Furthermore, some cytokines, including IL-6, IL-10, and TNF-α, have been described as biomarkers related to severe SARS-CoV-2 infection [139,140,141,142]. According to bibliometric analysis published by Xu et al. [108], the “cytokine storm” is one of the main pathogenesis mechanisms of SARS-CoV-2 virus-induced COVID-19. Therefore, inhibiting the cytokine storm may be a good approach toward combating COVID-19 infection [143]. As mentioned earlier, LF exerts immunomodulatory actions by inducing the T-cell activation, suppressing the levels of cytokines including IL-6 and TNF-α, up-regulating ferroportin and transferrin receptor 1, and down-regulating ferritin, pivotal actors of iron and inflammatory homeostasis [70,132,144] (Table 4). Consequently, LF inhibits intracellular iron overload, an unsafe condition enhancing in vivo susceptibility to infections, as well as anemia of inflammation [70]. In the study of pulmonary acute respiratory distress syndrome (ARDS) in granulomatous inflammation model [74], it was found that LF can reduce or eliminate cytokine excess and pulmonary pathological features caused by *Mycobacterium tuberculosis* [145,146]. In addition, LF was also able to diminish hyperacute immunopathology developed in murine models of *Mycobacterium tuberculosis* infection [147,148]. LF inhibits SARS-CoV-2 infection in different cell models with multiple modes of action, including enhancing interferon responses [105,149] (Table 4). Overall, these recent research studies may make LF an exciting clinical candidate for the treatment or prevention of SARS-CoV-2 in the future.

### 3.5. Microbiota Modulation

The efficacy of LF as a selective modulator of the microbiome was confirmed in several tests in vitro, in animal models and in human studies [57,150,151]. LF successfully altered the microbiota of the gut by eliminating pathogenic microorganisms and increasing the beneficial bacteria, such as bifidobacteria and lactobacilli that restored the state of eubiosis and protected against the serious consequences of dysbiosis. Several studies showed that intact as well as the proteolytic fragments derived from LF selectively stimulate the growth of beneficial bacteria and act as a selective microbiota modulator thereby preventing the proliferation of pathogens that cause diarrhea, such as *Salmonella* or rotavirus [59,151,152].

Even though COVID-19 is mainly a respiratory disease, there is accumulating evidence indicating that the gut is involved as well. According to a recent meta-analysis of 60 studies comprising more than 4000 patients, about 18% of COVID-19 patients suffer from gastrointestinal complaints like diarrhea, nausea/vomiting, or abdominal pain [7]. Associations between gut microbiota, ACE2, and inflammatory markers in COVID-19 patients indicate that the gut microbiota is involved in the extent of disease severity possibly via modulating host immune responses. Furthermore, the gut dysbiosis after disease resolution could contribute to lasting symptoms, emphasizing a need to understand how the gut microbiota are involved in COVID-19 [48,49,153,154]. A recent proof of concept trial showed that a well-studied probiotic, *Lactobacillus* LGG, is correlated with extended time to development of COVID-19 infection, decreased incidence of symptoms, and changes in the gut microbiota when used within a week after exposure [155]. If LF induces a similar response in COVID-19 patients needs further research.

### 3.6. Intestinal Barrier Function

LF is able to promote cell proliferation and differentiation in the gastrointestinal tract. High concentrations of LF stimulate intestinal epithelial cell proliferation, whereas low LF concentrations stimulate intestinal differentiation [156]. Healthy gut epithelial cells act as a transcellular barrier, while the tight junctions between cells provide a paracellular barrier against translocation of microbes and substances from the lumen [59]. In a preclinical model, oral administration of LF significantly enhanced tight junction protein expression (Claudin-1, Occludin, and ZO-1) and lowered intestinal permeability, suggesting an improvement in intestinal barrier function [131,157,158]. In humans, oral recombinant human LF supplementation reduced a drug-induced increase in gut permeability and hence may provide a nutritional tool in the treatment of permeability-associated illnesses [159]. An intact gut epithelial barrier is critical for normal physiological functions and decreasing bacterial translocation. These data suggest a role for LF in supporting gastrointestinal health.

Even though SARS-CoV-2 is thought to mainly transmit via lung droplets, it can invade enterocytes, causing symptoms and acting as a reservoir. Gut symptoms can be the first clinical sign in children [160]. It recently has been shown that the epithelial barrier function is compromised in COVID-19 patients leading to an inflammatory response [161]. Moreover ACE2, the main SARS-CoV-2 receptor, contributes to the maintenance of epithelial barrier function and its expression is negatively correlated with SARS-CoV-2 virus load [48,162]. As mentioned earlier, LF stimulates the growth of beneficial gut microbiota and the proliferation/differentiation of enterocytes with direct anti-inflammatory activities [150,156]. These strengthen mucosal immunity and the gut epithelial barrier. These LF effects have not been examined in COVID-19 patients, but this could be considered as interesting approach in the battle against SARS-CoV-2 infection, as they have positively affected recovery from other coronaviruses.

## 4. Lactoferrin Intervention Studies in COVID-19 Patients

Considering the results obtained from (pre)clinical studies, several human trials are published and 9 are in progress to investigate the anti-SARS-CoV-2 actions of LF for both prevention and adjunct therapy of COVID-19 [13]. First of all, an ex vivo study reported that LF may inhibit SARS-CoV-2 entry into nasopharynx and oral mucosa of COVID-19 patients by either directly binding to the viral particles or blocking the virus (co-)receptor present on the host cell [163]. This suggests that LF may already play a role in the early stage of infection. The evidence collected so far from 4 human studies is limited [69,85,164,165] (Table 5). However, some encouraging results are reported, especially in relation to the duration of the infection and the decrease of symptom severity. The interventions included LF and recombinant human LF. In 3 studies LF was encapsulated, because LF is a protein and subject to digestion in the gastrointestinal tract after oral administration. Two studies compared non-protected LF with encapsulated LF [132,166]. These studies indicate that encapsulation of LF improves the absorption.

A prospective observational study in 75 COVID-19 patients in Spain demonstrated that the combined oral administration of liposomal LF and zinc solution for 10 days allowed a complete and prompter recovery of all treated patients within the first 5 days of treatment. The same treatment, but at a lower dose seems to exert a potential preventive effect against COVID-19 in healthy people directly related to the affected patients [164] Both Campione et al. and Rosa et al. reported reduction in symptoms and shortening of illness duration by about 13 days when administering 200–1000 mg/d encapsulated LF, or 1000 mg/d liposomal and 16 mg/d intranasal LF in COVID-19 patients from Italy [69,165]. Furthermore, Rosa et al. showed that a significant correlation existed between age and effectiveness of LF in reducing the days of symptoms. The effectiveness of this treatment on symptom resolution was progressively higher in parallel with increasing age [165]. This fact could be associated with the hormonal control of human LF synthesis that decreases with age [168]. Elderly and in particular those suffering from Alzheimer’s disease show lower salivary human LF levels [126,169]. A few immunological outcomes improved within the LF-supplemented group (IL-6, D-Dimer), but others did not (TNF-α, IL-10, adrenomedullin) [69]. These findings are promising as monitoring of the course of COVID-19 infection revealed high levels of IL-6 and IL-10, but not always TNF-α, as markers of morbidity and mortality of patients [142]. In contrast, an Egyptian trial showed no significant differences regarding reduction in symptoms and immune parameters between 54 COVID-19 patients with mild-to-moderate symptoms receiving approved Egyptian COVID-19 management protocol and patients receiving the same treatment plus a non-encapsulated LF with a dosage of 200 mg/d [167]. This negative outcome might be due to the limitations of the trial: short duration of treatment (7 days) and/or limited sample size (18 patients/group). A recent systematic review covering studies of a diversity of viral respiratory tract infections, including SARS-CoV-2 concluded that LF (dosage range from 200–1000 mg/d) may help to decrease symptom duration and severity in SARS-CoV-2 infections, although the results between included studies are inconsistent according to the authors [85]. Further studies with larger samples as well as longer-term trials to understand the role of LF in treating SARS-CoV-2 are required. 

It is of note that 9 ongoing trials are registered in the WHO international trial registry platform focusing on LF intervention in COVID-19 patients (Table 6). Therefore, new data will become available in the future, allowing a more conclusive judgement on LF’s potential benefits as supporting nutritional intervention. Overall, even if larger clinical trials are required, these recent intervention trials indicate that early treatment of COVID-19 patients with LF could be one of the best strategies to avoid the disease onset, progression and severity, especially in patients with an advanced age. These results combined with the high tolerance consistently shown in the studies, makes the LF supplementation an interesting intervention for further investigations.

## 5. Information Gaps and Research Opportunities

Key questions that are not yet understood include determining the dose required, the best form of LF to use (e.g., intact or LF peptides, (non-)encapsulated, (minimally) processed, and capsules, liquid or powder) and the best route of administration (oral or intranasal). In the human intervention trials only intact LF has been used so far in a dose ranging from 200 mg to 1000 mg. The formats vary from capsules with LF being encapsulated into liposomes or non-encapsulated to a spray form. Thus far trials with encapsulated LF have shown promising results, whereas the one trial using non-encapsulated LF did not. Increasing LF’s stability and/or its absorption might improve clinical outcome. Encapsulation might be one way, but other food options are also possible like combining it with milk-derived osteopontin in a dairy drink [170]. Two routes of administration were tested, oral and intranasal. However, it is too early to draw any conclusions on the preferred route based on current evidence.

Most of the evidence being cited in support of LF’s ability to deal with COVID-19, is based on preclinical research. The human trials that have assessed LF as a COVID-19 treatment, have some limitations (short duration of treatment, limited sample size, no placebo group and a low dosage). Further work needs to be done to truly understand how LF can be applied in varying COVID-19 cases. It is important to note that gold standard clinical studies are required to generate results that have high confidence. Regulators around the world tend to set a high threshold for claims that a product can maintain health, treat or cure a disease. This means that for regulators to approve a health claim or a disease reduction claim on LF, they would likely require evidence from multiple large randomized-placebo-controlled clinical trials relevant to the local population. The population to be studied would have to be clearly defined, because clinical findings in one subset of the population (e.g., elderly) will not necessarily translate to another part of the population (e.g., young adults). Although LF displays the many bioactivities (anti-viral, anti-bacterial, immune-supporting, microbiota- and barrier function modulation) described in this review, there is currently a lack of robust human clinical research to suggest it can specifically prevent or cure COVID-19. However, 9 trials are ongoing which no doubt will generate more data in the future, allowing a more conclusive judgement on LF’s potential benefits as supporting nutritional intervention.

Whilst it is understandable that consumers want to proactively seek solutions for themselves, it is important that they do not resort to yet unproven COVID-19 treatments. Although the recent vaccines provide hope for reducing the risk and severity of infections, the authors strongly reiterate the advice of many governments and health organizations that there is currently no concrete scientific evidence to suggest that there is any treatment that is guaranteed to specifically prevent or cure COVID-19.

In order to further build the science behind bovine LF’s effect on the immune system and/or pathogen infection, future well-designed randomized placebo-controlled studies could focus on middle-aged or elderly people to see if LF either increases a vaccine response or if it ameliorates a response to a “controlled stressor” (inactivated pathogen) or a real SARS-CoV-2 infection. Middle-aged or elderly volunteers are selected as target population because LF was shown to be more effective in the older population than the younger one. The vaccine response model may shorten the timeframe of the trial and decrease the number of participants needed. It also decreases the risk that volunteers will not conform to the study specifications or will drop out altogether. By inducing symptoms or disturbing the physiological balance through the introduction of a controlled stressor, it is possible to speed up validation, while requiring fewer participants. With this approach, it might be possible to get a health benefit of LF substantiated more quickly and cost-efficiently.

## 6. Conclusions

**Figure 3:**
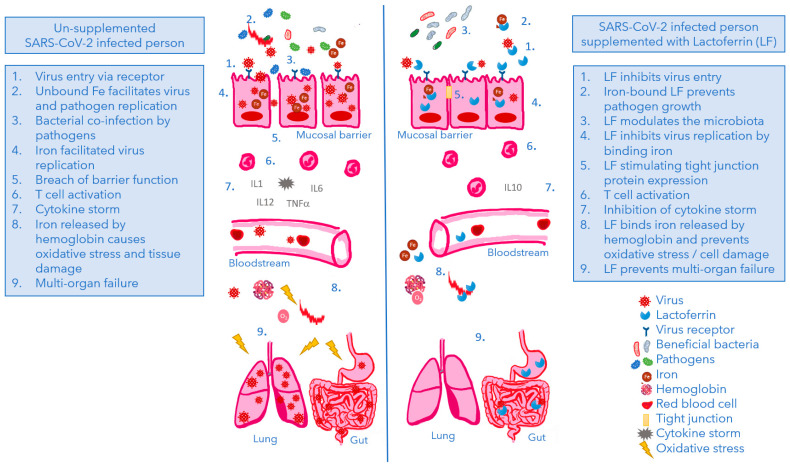
Model illustrating the different stages of a SARS-CoV-2 infection in un-supplemented (left panel) and in LF-supplemented (right panel) COVID-19 patients. Based on the here presented data, LF protects the host from viral infections by prevention of binding and entry of SARS-CoV-2, and sequestering iron needed for viral replication. Furthermore, it indirectly affects virus attacks by having anti-oxidant effects and immune-, microbiome-, and intestinal barrier-modulatory characteristics.

The existing evidence suggests daily intake of LF may have protective effects against SARS-CoV2 infection as is illustrated in Figure 3. Especially, this review highlights the multiple possible physiological effects of LF (iron binding, anti-viral, anti-bacterial, immune-supporting, microbiota- and barrier function modulation) in the battle against COVID-19. LF intake may protect the host from viral infections by prevention of binding and entry of various SARS-CoV-2 variants and sequestering iron needed for viral replication. Furthermore, it indirectly affects virus attacks by having immune-, microbiome-, and intestinal barrier-modulatory characteristics. These six physiological effects of LF mostly support gut- and lung health of people at risk or suffering from COVID-19. Most of the scientific evidence originates from preclinical and ex vivo studies. However, the results of the human intervention studies support the preclinical findings and demonstrate a potential anti-viral and immune-supportive effect of 200 mg–1000 mg LF involving multiple sites of the human immune system, covering the innate and adaptive immune system. In particular LF influences: (1) expression of pivotal biomarkers of COVID-19 like IL-6; (2) B-cell differentiation, total T-cell activation, T-helper cell activation and cytotoxic T-cell activation, (3) intestinal permeability strengthening the intestinal barrier function, (4) incidence and length of common and summer colds as an indication of an improved defense against viral infections in the respiratory tract, which again is also supported by (5) the effects of LF on the length and severity of symptoms in COVID-19 patients. However, the available evidence needs to be expanded with well-designed human intervention studies in order to confirm its protective effects against SARS-CoV-2 and determine the dose required, alongside the best form of LF to use (e.g., intact or LF peptides, encapsulated or non-encapsulated), and to establish the best route of administration (oral or intranasal). From results to date of human studies measuring immune responses, it is not possible to provide an indication what part of the immune system is effective in the protection against viral infections of the respiratory tract. However, 9 new intervention studies are on their way to further study the protective role of LF in the battle against COVID-19. The near future may therefore provide new data behind the potential benefits of LF supplementation as a supporting nutritional measure.

## Figures and Tables

**Table 1:** Physiological effects as part of COVID-19 pathogenesis (left column) and counteracting iron-related effects of LF clinically demonstrated in COVID-19 patients or other populations (right column).

COVID-19 Pathogenesis	LF’s Iron-Related Effect
Iron deficiency risk increases	LF increases iron absorption thereby lowering the risk of iron deficiency ^2^
Ferritin and IL6 levels increase	LF decreases ferritin and IL6 levels ^1^
Intracellular iron overload increases viral replication	LF decreases the intracellular iron level ^2^ resulting in reduced viral replication ^1^
Virus attacks hemoglobin leading to iron and oxygen release thereby inducing oxidative stress	LF chelates iron thereby reducing oxidative stress ^2^

^1^ Effect detected in COVID-19 patients or ^2^ in other populations.

**Table 2:** Physiological effects as part of COVID-19 pathogenesis (left column) and corresponding anti-viral effects of LF (right column).

COVID-19 Pathogenesis	LF’s Anti-Viral Effect
SARS-CoV-2 attaches to HSPG, concentrates on the cell surface and subsequently binds to ACE2 for cell entry	LF blocks binding of SARS-CoV-2 to HSPG in an ACE2 and iron independent manner
	LF inhibits virus infection of different SARS-CoV-2 variants. Its efficacy resides in LF’s *N*-terminus
Virus replication is enhanced by intracellular iron. The virus hijacks the cell’s stress response system involving sigma receptors	Iron-binding LF inhibits virus replication and can complement specific anti-viral drugs

**Table 3:** Physiological effects as part of COVID-19 pathogenesis (left column) and corresponding anti-microbial effects of LF (right column).

COVID-19 Pathogenesis	LF’s Anti-Microbial Effect
About 72% of COVID-19 patients in health care settings received antibiotics	LF inhibits growth of gram-negative and gram-positive bacteria, fungi, and protozoa
Prevalence of bacterial co-infection is ~7% and of secondary bacterial infection is ~14%	LF selectively inhibits pathogens, whereas it does not inhibit beneficial microbes
The oral cavity plays an important role in SARS-CoV-2 infection and transmission	LF is naturally present in the oral cavity providing microbial homeostasis
Elderly are more susceptible to (severe) COVID-19	LF levels in saliva decrease with age, leading to a dysbiosis and susceptibility to disease

**Table 4:** Physiological effects as part of COVID-19 pathogenesis (left column) and counteracting immune effects of LF (right column).

COVID-19 Pathogenesis	LF’s Immune Effect
Prominent early features of COVID-19 include a pronounced reduction in B cells important in defense against SARS-CoV-2	LF has a profound modulatory action by the differentiation of immature B-cells into efficient antigen presenting cells
Immune system may over-react sending in neutrophils, T-helper- (CD4) and cytotoxic T-cells (CD8) that release pro-inflammatory cytokines, especially IL-1 and IL-6	LF increases total T-cell activation, T-helper cell activation and cytotoxic T-cell activation, and suppresses cytokines levels including IL-6 and TNF-α
The ‘cytokine storm’ damages normal lung cells more than the virus it targets leading to acute respiratory distress syndrome (ARDS)	In a model of pulmonary acute respiratory distress syndrome (ARDS) in granulomatous inflammation, LF can reduce pulmonary pathological features
Some cytokines, including IL-6, IL-10, and TNF-α, have been described as biomarkers related to severe SARS-CoV-2 infection	LF supplementation decreases levels of cytokines including IL-6 and TNF-α, and increases IL-10

**Table 5:** LF intervention trials in COVID-19 patients.

Reference	Title	Dose (mg/d)	Intervention	Population	Country	Main Outcomes
Serrano et al., 2020 [164]	Liposomal Lactoferrin as Potential Preventative and Cure for COVID-19	120–180 and 60–90	10 days	75 patients + 256 family members	Spain	Faster recovery time and symptom relieve from e.g., weakness, loss of smell and taste, cough and muscular pain.
Algahtani et al., 2021 [167]	The Prospect of Lactoferrin Use as Adjunctive Agent in Management of SARS-CoV-2 Patients: A Randomized Pilot Study	200	7 days	54 patients (mild-moderate)	Egypt	No statistically significant difference among studied groups regarding recovery of symptoms or blood/immune parameters
Campione et al., 2021 [69]	Lactoferrin as Antiviral Treatment in COVID-19 Management: Preliminary Evidence	1000 + 16	30 days	92 patients (mild-moderate)	Italy	Reduction in symptoms and shortening of illness duration by about 13 days. Some COVID19-biomarkers improved (IL-6, D-Dimer, ferritin)
Rosa et al., 2021 [165]	Ambulatory COVID-19 Patients Treated with Lactoferrin as a Supplementary Antiviral Agent: A Preliminary Study	200–1000	50 days	121 patients (mild-moderate)	Italy	The time required to achieve a negative SARS-CoV-2 PCR result was significantly lower. The effectiveness on symptom resolution was progressively higher in older adults.

**Table 6:** Registered LF intervention trials in COVID-19 patients in WHO international trial registry platform.

Trial ID	Title	Age	Country	Primary Outcome
NCT 04412395	Clinical Assessment of Oral Lactoferrin as a Safe Antiviral and Immunoregulatory in Treating COVID-19 Disease	18–80	Egypt	Survival rate; Rate of disease remission; The number of patients with PCR negative results.
NL9742	Lactoferrin in the treatment of Long COVID	18–60	The Netherlands	Do fatigue symptoms diminish faster with the use of lactoferrin combined with usual care compared to usual care solely?
CTRI/2021/ 12/038672	Clinical trial on Mild and Moderate COVID-19	>18	India	Change from baseline in CRP, D-dimer and other biomarkers; Proportion of patients and time to progress from mild to critical grade; Reduction in viral shedding; Improvements in COVID-19 symptoms
NCT 04713735	Impact of Lactoferrin vs. Placebo on Respiratory Tract Infections	>55	USA	Number of Respiratory Tract Infections
NCT 04847791	Lactoferrin in COVID-19 Hospitalized Patients	18–99	Italy	Intensive care unit hospitalization rate; death; proportion of discharged patients; National Early Warning Score (NEWS)
NCT 04621149	An Outpatient Study Investigating Non-prescription Treatments for COVID-19	20–70	USA	Reduction in Participant Symptoms of COVID-19
PER-064-20	Lactoferrin for Prevention of COVID-19 in Health Care Personnel	18–59	Peru	Serology (IgM or IgG) or RT-PCR for COVID-19; Number of COVID-19 infections
NCT 04427865	Utility of Lactoferrin as a Preventive Agent for Healthcare Workers Exposed to COVID-19	18–65	Egypt	Incidence of SARS-CoV-2
NCT 04421534	Utility of Lactoferrin as an Adjunct Therapeutic Agent for COVID-19	18–65	Egypt	Time to clinical improvement
